# Choice of transformation for modelling non-linear continuous biomarkers

**DOI:** 10.1186/1745-6215-12-S1-A20

**Published:** 2011-12-13

**Authors:** Deborah D Stocken, Lucinda J Billingham, Philip J Johnson, Nick Freemantle

**Affiliations:** 1 Cancer Research UK Clinical Trials Unit, University of Birmingham, Birmingham, UK; 2 Primary Care and Population Health, University College London, London, UK

## 

Identification of prognostic and predictive biomarkers is important for targeting treatments to patients and for the design and analysis of randomised controlled trials. Cox proportional hazards modelling is a standard method for assessing prognostic value of clinical biomarkers where time to occurrence of an event is the primary outcome of interest. An important issue in the analysis of prognostic factors is the functional form of the relationship between the factor and outcome specifically for continuous covariates. Continuous covariates are often simplified assuming a linear relationship with log-hazard or dichotomisation which may be inappropriate leading to loss of efficiency in statistical tests, bias and incorrect conclusions. The effects of important prognostic biomarkers may go unrecognised due to simplistic assumptions made in statistical modelling.

Two polynomial based strategies, restricted cubic splines and fractional polynomials, are compared directly for determining the functional form of non-linear relationships between prognostic biomarkers and survival using two real datasets from randomised controlled trials in advanced pancreatic cancer and cardiac surgery. Fractional polynomials are an extended family of curves including non-integer and negative power terms. Spline functions are piecewise polynomials connected across intervals of a variable constrained to meet at the ‘knots’.

Multivariable models were constructed based on Cox proportional hazards regression using backward elimination. Internal validation to directly compare the fit of the restricted cubic spline and fractional polynomial strategies was carried out by calculating the sampling distribution of the difference in AIC between the models using nonparametric bootstrap analyses. Further analysis recalculated the univariate fractional polynomial transformation within each bootstrap resample to compare directly against a 5-knot restricted cubic spline. The influence of the size of the bootstrap samples was also investigated.

The fitted functions generated by splines and fractional polynomials were similar resulting in comparable models. The methods are generally different in the extremities where there is often a paucity of data. Larger differences were seen between the two methods when sample sizes were reduced due to the reduced power to detect small effects but also to detect nonlinearity.

Multivariable fractional polynomial transformations are an alternative approach to restricted cubic spline transformations for multivariable model building of continuous biomarkers with non-linear relationships with outcome.

